# Structural insights into HRI kinase activity and inhibition

**DOI:** 10.1042/BST20250541

**Published:** 2026-05-15

**Authors:** Min Cao, Glenn R. Masson

**Affiliations:** 1 MRC Protein Phosphorylation and Ubiquitination Unit, Faculty of Life Sciences, University of Dundee, Dundee, U.K.; 2 Division of Cancer Research, Faculty of Heath, University of Dundee, Ninewells Hospital, Dundee, U.K.

**Keywords:** DELE1-CTD, eIF2alpha, haem, HRI, ISR, kinases

## Abstract

Haem-regulated inhibitor (HRI) is emerging as a potential therapeutic target in several disease areas, including cancers such as multiple myeloma and neurodegenerative disease. As one of four related mammalian kinases that phosphorylate the mRNA translation machinery substrate eIF2α, it has a central role in sensing several disparate proteotoxic stresses and preventing subsequent rounds of protein production. In this review, we will examine the latest research on the structural and molecular basis of HRI inhibition and activation, examining both regulatory biological interactions with proteins and cofactors. What emerges is that despite almost 75 years since the first identification of HRI, we still know remarkably little about how this kinase functions and is regulated.

## Introduction

Haem-regulated inhibitor (HRI) (gene *EIF2AK1*) was the first of the four eIF2α kinases identified in the 1960s and 1970s through the pioneering work of (among others) RS Ranu, N.K. Gupta, Irving M. London, Tim Hunt, and Richard Jackson, all of whom were investigating the mechanism of protein synthesis inhibition in rabbit reticulocytes [1–6]. Using rabbit reticulocyte lysates as an early model system of *in vitro* translation, systematic testing with additives such as haem and double-stranded RNA allowed these scientists to pick apart the basis of what would eventually be recognised as the Integrated Stress Response (ISR). In these early experiments HRI was identified as a* haem-regulated inhibitor of protein synthesis*—although it would only subsequently be determined that HRI was a kinase, with later studies uncovering the related kinases PKR (Protein Kinase R), PERK (PKR-like endoplasmic reticulum kinase) and GCN2 (general control non-derepressible 2), all of which are themselves highly sought-after drug targets and key proteins controlling the cell’s response to unique stresses.

The discovery of these four kinases produced a generalised view of the ISR: when a cell experiences a diverse set of stresses, the ISR is activated to restore homeostasis. Autoinhibited kinases sense the stress, become activated, autophosphorylate, and then phosphorylate eIF2α (Eukaryotic translation initiation factor 2A) on Ser51, which globally suppresses protein translation while selectively enhancing translation of stress-responsive transcripts such as ATF4 (activating transcription factor 4). Activation of ATF4 allows for the up-regulation of stress-mitigating genes, tailored to the upstream initiating stress, which are expressed during global mRNA translation repression due to a short upstream open reading frame in their 5′ untranslated region [7]. The ISR has emerged as a crucial mediator of proteotoxic stress and major target for drug discovery in a diverse range of disorders and diseases.

In the intervening years since the discovery of HRI, it may be safe to say that HRI took a back seat to the other ISR kinases, with GCN2 and PERK especially being investigated in their roles in tumourigenesis and cell growth, while HRI’s role was thought largely to be a regulator of haemoglobin biogenesis in red blood progenitor cells [8]. This excellent research was spearheaded by Jane-Jane Chen of MIT, whose dedicated research on HRI for over 20 years uncovered critical aspects of how HRI is regulated [9]. This research laid the foundation for the recent discoveries related to HRI function, where HRI was shown to be expressed in many tissues [10], notably in epithelial cells, where it partakes in additional roles such as mitochondrial stress sensing [11–14] and sensing proteotoxic stress through unfolded proteins and proteasome inhibition [15,16]. In this review, we will address the known post-translational modifications, binding partners, cofactors and ligands that regulate HRI, and relate them to potential structural changes in HRI.

## HRI structure

Human HRI is a 630 amino acid protein which has a high degree of conservation in higher eukaryotes, with mice and rats sharing 99% sequence identity. Phylogenetic analysis has suggested that HRI originated from Platyhelminthes/Nemathelminthes (flatworms/nematode worms) [17], but *Schizosaccharomyces pombe* (fission yeast) also carries HRI-like protein(s) [18] with 28% sequence identity (*Saccharomyces cerevisiae*, however, hosts only a single eIF2α kinase, Gcn2, which is thought to be the ancestor of the other members of the eIF2α family [17,19]). Of note, in the literature, several articles erroneously state that *S. pombe*’s HRI (Hri1p/Hri2p) is insensitive to haem; this is not the case [20]. Zhan *et al.* demonstrated that Hri1p/2p are as sensitive to haemin as rabbit (*Oryctolagus cuniculus*) HRI [18].

**Figure 1:**
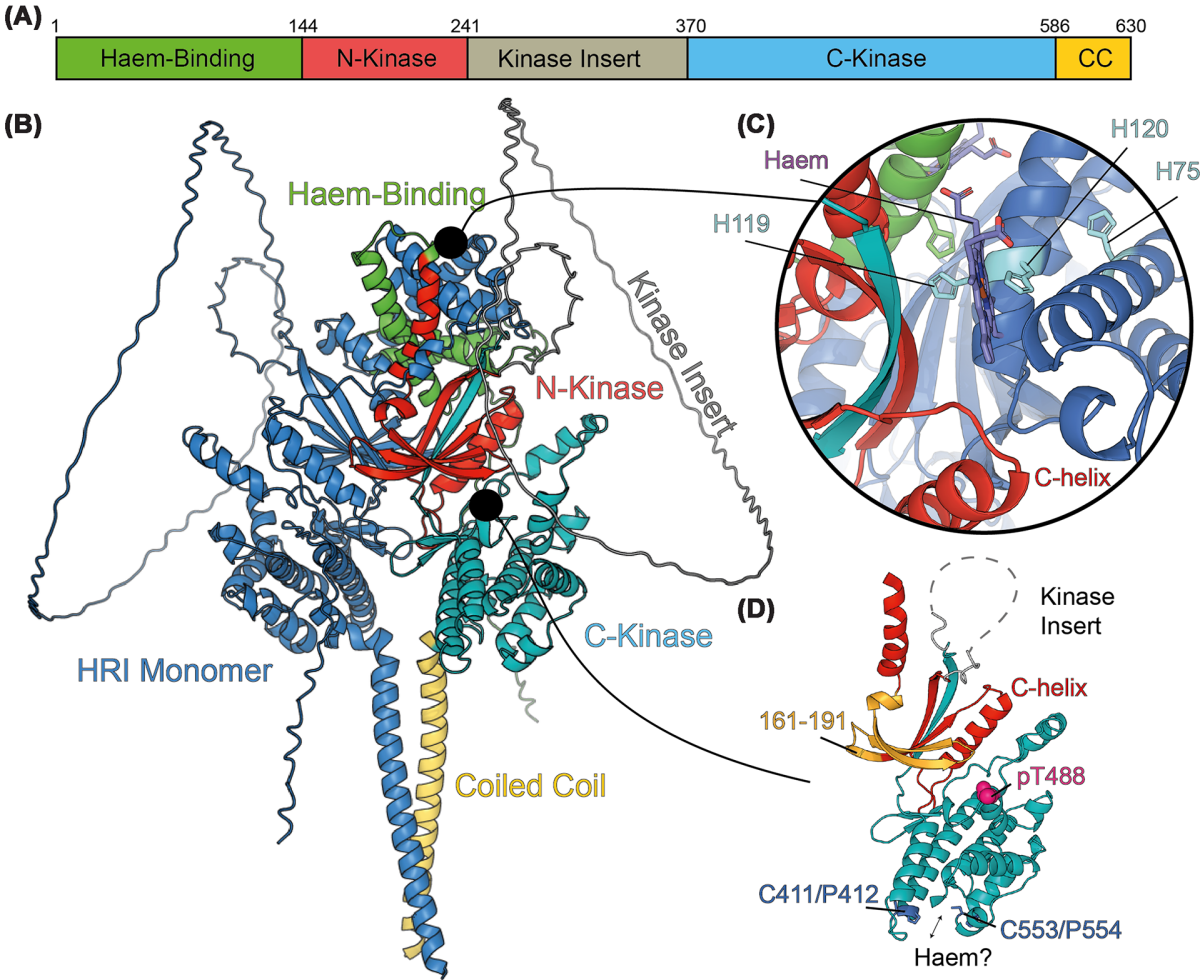
Predicted structure of HRI and potential basis of haem inhibition (**A**) Domain architecture of HRI, with haem-binding domain (HBD), N-kinase, kinase insert, C-kinase, and coiled coil (CC) domains. (**B**) AlphaFold 3 prediction of HRI dimer, with no haem bound. One monomer is coloured entirely blue, while the other is coloured using the same system as shown in panel (A). (**C**) Insert of the predicted haem-binding site in the HBD highlighting key residues. H120, H75, and H119 are shown with side chains in light blue. Binding prediction conducted using AlphaFold 3, co-folding with 4 haem molecules and 2 HRI polypeptides. (**D**) Isolated kinase domain (produced from AlphaFold 3) showing overall architecture. Residues 161–191 are the predicted site of p58^IPK^ binding. pT488 is a critical autophosphorylation residue required for activation of HRI. Finally, the four other residues predicted for haem binding (C411/P412, C553/P554) are also shown at the bottom of the C-kinase domain, coloured blue.

Currently, there are no published structures of HRI, either intact or from any of the isolated domains. Like all the eIF2α kinases, HRI exists as an obligate homodimer, however, the domain organisation of HRI is unique, with an N-terminal “haem binding” domain (1–144), followed by a split kinase with N- (144–241) and C- (370–586) lobes coming together to form a kinase fold (see Figure 1). Finally, there is a small, likely coiled-coil domain between residues 586 and 630 [21,22]. Notably, all three of the other ISR kinases (PKR [23], PERK [24] and GCN2 [25,26]) have had their kinase-domain structures determined by X-ray crystallography, frequently in highly truncated forms, often with inactivating mutations. The kinase domain of HRI is most similar to GCN2, but several critical residues for HRI regulation are unique to HRI (see below), and so the absence of an HRI kinase domain structure limits our understanding of how HRI may be regulated structurally.

Note that in some publications, there has been some confusion about the number of kinase domains present in HRI due to the N- and C-lobes being labelled as Kinase 1 and Kinase 2, respectively; to avoid this confusion, we use the N- and C-kinase nomenclature. Within HRI, there are unstructured segments identified through both prediction [21] and biophysical means, including hydrogen-deuterium exchange [22], including the first ∼70 amino acids, the kinase insert loop of ∼240 to ∼370, and the rather extended kinase activation loop of 461–495. These sections remain unstructured while HRI is dimerised (as measured by Hydrogen Deuterium Exchange Mass Spectrometry (HDX-MS), size-exclusion chromatography and mass photometry) [22], suggesting that they do not partake in any dimerisation-induced folding event. This dimerisation is likely critical to the large-scale autophosphorylation event which accompanies HRI activation (as has been shown in the other eIF2α kinases also) [22,27,28]. One critical autophosphorylation residue is T488 (see Figure 1D), found within the activation loop of HRI, and has been found (in the mouse paralogue) to be critical for activation and autophosphorylation [29].

In the absence of empirically derived structures, we can use AlphaFold predictions of HRI dimers to possibly glean some insight into the nature of the HRI structure [21,22]. AlphaFold 3 produces a back-to-back kinase domain structure (see Figure 1B), with the HBD sitting as a nest of alpha-helical structures atop the N-lobe of the kinase domain, and a large pair of helices extending down from the C-lobe of the kinase domain [22,30]. It is worth noting that the AlphaFold predicted modelling metrics (the predicted template modelling score and the interface predicted template modelling score) are both 0.5, meaning there is a medium-to-low confidence overall in the fold, with parts of the HBD, the N-terminal and kinase insert disordered sections, alongside the C-terminal helices all having poor local prediction scores too, so the structure presented should be assessed with some level of caution.

## Down-regulation of HRI

A table summary of predicted regulators of HRI is shown in Table 1. The most well-characterised binder and inhibitor of HRI is haem (heme). Haem has been shown to inhibit both autophosphorylation and the phosphorylation of the substrate eIF2α, with two likely binding sites: one in the C-lobe of the kinase domain around residues C411/P412 and C553/P554 (in mouse, C409/P410 and C550/P551, with which the study was conducted), and another site in the HBD around H75 and H120 (same numbering in human and mouse) (see Figure 1C,D) [20]. It should be noted that the vast majority of biochemical experiments have been conducted with the haem analogue Haemin (hemin), rather than haem itself, which is present in cellular systems in four possible varieties (A/B/C/O) [31]. Haemin and the four types of haem differ by their porphyrin ring, and this may impact each variant’s ability to interact with HRI.

**Table 1:** Regulators of HRI

**Inhibitors**		
	Mechanism	Site of regulation on human HRI
Haem	Direct binding, likely two haems per HRI monomer [20,32], IC^50^ of bacterially produced HRI ∼ 2–5 μM [20–22,27].	H75, H119/H120, C411/P412, C553/P554 (by homology) [20].
p58^IPK^ (?)	Not directly shown, but p58ipk binds and inhibits the other eIF2α kinases—HRI was not tested [36].	Likely ∼161–191 (by sequence homology with GCN2).
SIFI	Ubiquitination of HRI and degradation, possibly in complex with DELE1-CTD activator [37,38].	Likely K92 or K100 in the haem-binding domain [38].
**Activators**		
DELE1^CTD^ [11–14,39]	Possibly mediated by DELE1^CTD^ oligomerization, binds HRI promotes its trans-autophosphorylation, may displace haem.	???
BAG3/Hsp70 [40,41]/ HSPB8 [16] (?)	BAG3 recruits HRI into the Hsp70-BAG3; BAG3’s CaMKII-dependent phosphorylation.	???

**Figure 2:**
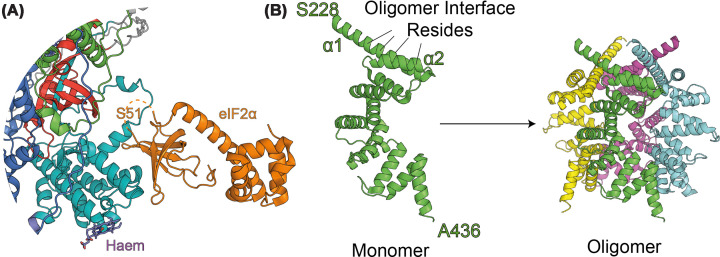
Substrates and potential activators of HRI (**A**) Model structure of HRI–eIF2α complex using AlphaFold 3 (HRI) aligned with an x-ray crystallography-derived structure of the PKR kinase domain in complex with eIF2α (PDB: 2A1A). Also docked, using AlphaFold 3, is a haem molecule at the proposed C411/P412 and C553/P554 binding sites. (**B**) Structure of DELE1^CTD^, residues S228–A436. This structure, derived from a construct DELE1 225–515, can readily oligomerise, and this is largely dependent on residues found around 240–250 in the α1 and α2 helices (PDB: 8D9X).

Rigorous experiments conducted by Rickett *et al*. indicated that haemin was likely functioning through noncompetitive inhibition with respect to ATP and eIF2α [21]. Hydrogen deuterium exchange mass spectrometry analysis of haemin binding suggested there are very large conformational changes throughout HRI when bound to haemin, not least in the disordered kinase insert, the HBD and the kinase domains. This suggests a complex structural reorganisation prevents HRI autophosphorylation and activation, rather than the simple competitive model in which haem binds to the C-terminus of the kinase domain and prevents eIF2α binding [22]. Another piece of evidence which would refute a competitive model would be the PKR/eIF2α co-structure [23] (PDB: 2A1A), which shows that any haem molecule bound at C411-P412/C553-P554 would be unlikely to impede eIF2α interaction with HRI (see Figure 2A).

What is also not clear is the relative role that the kinase domain haem binding site and the HBD haem binding site play in the structure and regulation of HRI. In the Rafie-Kolpin *et al.* study, removal of the HBD lowered activity of HRI and lowered haem sensitivity (*i.e.,* haem became a less potent inhibitor) [32]. In the same study, a construct (231–420) which contains the C411/P412 pair was also shown to bind to haem [32], but subsequent mutation of that cysteine to serine (C409S, mouse numbering), although it ablated haem interaction, appeared to have no effect on haem inhibition, suggesting this haem-binding site does not play a role in kinase activity regulation [20]. The *S. pombe* HRI paralogues, which are sensitive to inhibition by haemin, also have the histidine pair (H105/H106 SpHri1p, H98/99 SpHri2p [18]—again, this has been misrepresented in previous publications), although they have no obvious cysteine–proline pairs in the kinase domain. Using AlphaFold 3, it is possible to dock haem into both the kinase and HBD sites of Human HRI (see Figure 1C). Although only a prediction and should be treated with caution, it is interesting that the HBD site haem binds in a pocket formed at the interface of one HRI chain’s H119/H120 site and the N-lobe of the kinase domain of another HRI chain, close to the C-helix, potentially facilitating regulation of the kinase domain, rather than at the disordered kinase insert.

One note is that many studies, including the authors’ [22], use (typically human) HRI recombinantly expressed and purified from *Escherichia coli* [21]. While *E. coli* HRI is dimeric and is an active enzyme capable of phosphorylating eIF2α [21,22], it does display different haem interaction characteristics from mammalian-expressed HRI. Rabbit reticulocyte lysate-purified HRI is purified already bound to haem [33] and displays an IC_50_ for haem approximately 10-fold more potent than bacterially produced HRI (at ∼0.25 μM). As *E. coli* do not produce haem, it is worth considering that if haem binding has a structural role and binds co-translationally to HRI, and whether our data derived from bacterial HRI may be missing critical aspects of structural regulation of HRI.

There is a candidate protein inhibitor of HRI, protein p58^IPK^ (also known as DnaJC3 (DnaJ heat-shock protein (hsp) 40 homologue, subfamily C, member 3)) [34,35]. The p58^IPK^ protein is a tetratricopeptide repeat (TPR) protein that essentially acts as a co-chaperone of Hsp70, and previous work has shown that p58^IPK^ is a general eIF2α kinase signalling inhibitor, with immunoprecipitation experiments suggesting that p58^IPK^ stably interacts with GCN2, although whether this inhibition is truly through a direct interaction is not clear [36].

Finally, there is an additional inhibitor of HRI—the E3 ubiquitin ligase silencing factor of the integrated stress response (SIFI) complex—which terminates the ISR through degradation of HRI [37]. In a series of exhaustive and elegant papers, the work of Rapé group determined that the SIFI complex directly targets HRI for polyubiquitination and subsequent degradation after activation via mitochondrial damage [37,38]. Although it is not known for certain which residues are ubiquitinated, it is likely the lysine residues within a helix stretching from residues 85–101, found within the HBD, which are targeted by the complex.

## Activation of HRI

In parallel with the inhibitors described above, the predicted activators of HRI are listed in Table 1, among which DELE1 (DAP3-binding cell death enhancer 1), a reporter of mitochondrial damage, has been studied most extensively.

DELE1 has been reported to promote HRI activity following mitochondrial depolarisation or inhibition of oxidative phosphorylation. Under stress, DELE1 is cleaved at residue 142 by the inner-membrane metalloprotease OMA1, generating a C-terminal fragment that accumulates in the cytosol and facilitates phosphorylation of eIF2α via HRI activation [11–14]. Notably, deletion of either DELE1 or HRI markedly reduces ISR signalling under mitochondrial stress, highlighting the central role of the OMA1-DELE1-HRI axis [11–14]. More recent work indicates that DELE1 can also activate HRI independently of OMA1-mediated cleavage. Under basal conditions, newly synthesised DELE1 is imported into mitochondria and rapidly degraded by the matrix protease LONP1 (Lon peptidase 1) [11–14]. During intracellular iron deficiency, mitochondrial import is impaired, allowing full-length DELE1 to escape degradation and accumulate at the mitochondrial surface, where its C-terminal TPR domain (note the similarity to p58^IPK^) can engage cytosolic HRI and trigger ISR signalling [11–14]. Finally, in the work of Bi *et al.*, an alternative protease, HtrA2 (high-temperature requirement protease A2), may also play a role in DELE1 processing, creating an even smaller fragment of DELE1 in the cytosol which is dubbed DELE1-VS (for DELE "very short"), as opposed to DELE1-S created by OMA1/LONP1 [42].

Certain aspects of how HRI is activated by DELE1 are still not clear—such as whether DELE1 is in an oligomeric state or not. For example, when cleaved, Yang *et al.* have shown *in vitro* that DELE1 C-terminal domain (DELE1^CTD^) fragment is capable of oligomerising [39] (see Figure 2B) (PDB: 8D9X)—however, notably, the study was conducted with a fragment shorter (residues 225–515) than the likely *in vivo* fragment (∼142–515)—it is not known whether this longer fragment oligomerises. Additionally, it is not known whether this oligomerisation occurs within cells; mutations which disrupt the oligomerisation prevent ISR activation, but this may also affect protein stability and/or HRI binding. Furthermore, other studies have pointed to full-length DELE1 also being capable of activating HRI (or at least DELE1 that has not been cleaved by OMA1) [11], and in the Yang *et al.* study, full-length DELE1 was shown to be monomeric [39], suggesting that oligomerisation is not required. It may be that DELE1^CTD^ may also have a monomeric population; Yang *et al.* noted that on their cryo-electron microscopy grids there was a substantial amount of monomeric DELE1^CTD^ also visible, suggesting that the oligomers may be dynamic or concentration-dependent. In either case, DELE1, whether full-length or cleaved, appears important for efficient HRI activation—whether this is due to scaffolding HRI and facilitating trans-autophosphorylation [11–14] (in a manner perhaps similar to PKR binding to dsRNA [43,44]) remains to be seen.

Another outstanding aspect of DELE1-HRI activation is that it is not clear whether DELE1^CTD^ binding causes a release of haem—which would likely be necessary for HRI activation. Guo *et al*. conducted a biochemical assay to determine whether full-length DELE1 could activate HRI [13], which suggested that DELE1 only activates HRI in the presence of haem (or haemin)—suggesting that there is a mutual regulation between DELE1 and haem binding. In their structure of DELE1^CTD^ paper, Yang *et al.* conduct co-immunoprecipitation experiments using HRI truncations to determine the potential site of interaction between DELE1^CTD^ and HRI and localised the interaction site to the first 160 amino acids (*i.e.*, the HBD), which may point to a competitive mechanism of interaction at this site between DELE1^CTD^ and haem [39].

Beyond ISR induction, DELE1-HRI signalling has also been implicated in the negative regulation of PINK1-dependent mitophagy [14] (although it should be noted that in other studies DELE1-HRI activation has been suggested to promote mitophagy [45]). Both proposed models suggest that it is likely that up-regulated proteins downstream of ATF4 are the likely mediators of PINK1 rather than HRI itself. Collectively, these observations support a model in which DELE1 acts as a mitochondrial stress sensor, which is enriched in the cytoplasm to activate HRI and enable downstream ISR responses.

Distinct from DELE1-mediated HRI activation, HRI is also regulated by the co-chaperone and adaptor protein BAG3 (B-cell lymphoma 2 (Bcl-2)-associated athanogene 3), in complex with Hsp70 and/or HSPB8 in response to proteotoxic stress, particularly during proteasome inhibition and the accumulation of ubiquitinated misfolded proteins [15,40,41]. BAG proteins are co-chaperone proteins that contain the highly conserved BAG domain, which binds to and regulates the activity of the ATPase domain of chaperones such as Hsp70 [46,47].

The exact relationship between HRI, BAG3, Hsp70 and HSPB8 appears to be intricate and perhaps cell-type specific. The precise role this chaperone complex plays is still unclear, as this complex appears to be highly promiscuous with roles in numerous pathways, including mitophagy, autophagy, protein transport, G-protein coupled receptor signalling, and sarcomere integrity [48,49]. The BAG3-Hsp70/HSPB8 complex appears to have interactions with several stress-sensing kinases under proteotoxic stress [48–51].

Patel *et al.* suggested a model where a BAG3/Hsp70 complex interacts with HRI as a complex while HRI is in an inactive conformation, and HRI is activated when Hsp70 dissociates, or, while under proteotoxic stress, there is an accumulation of ubiquitinated Hsp70—suggesting that Hsp70 inhibits HRI [40]. Furthermore, Patel *et al.* found through pull-down assays that BAG3 and HRI co-elute, and that HRI also co-precipitates with polyubiquitinated proteins and that depletion of BAG3 prevented this association between HRI and ubiquitin, suggesting that BAG3 bridges this interaction may act as a scaffold. On the other hand, depletion of BAG3 via siRNA significantly reduced eIF2α phosphorylation during proteasomal inhibition—suggesting that BAG3 is necessary for HRI activation [40]. It is not clear exactly how HRI and BAG3 may interact. In their review of BAG3 signalling, Sherman and Gabai suggest that the M-domain (residues 213–302) of BAG3 may play a role, although there is little direct evidence supporting this [49].

Regulation of HRI activity by BAG3/Hsp70 might be further controlled by CaMKII (Ca^2+^/Calmodulin-dependent protein kinase II) [41]. In very thorough and elegant work, Zhang *et al.* demonstrated that under conditions of protein misfolding, CaMKII is activated and phosphorylates BAG3 on S173/377/386, and this enhances HRI activation, activating the ISR and thus suppressing the synthesis of misfolded protein. Two of the three phosphoserines, S377 and S386, sit within the PXXP domain of BAG3, pointing to a possible site of regulation with HRI.

Finally, another component of this chaperone complex which has been shown to regulate HRI activity is the small heat shock protein HSPB8 [15,16,47]. The research of Stephen Girardin highlighted that innate immune signalling can lead to the formation of toxic protein aggregates and demonstrated that HRI activity might link proteotoxic stress to innate immune pathways [15,16]. In two studies, the Girardin group found that the interaction between HRI and HSPB8 is critical for signalling downstream of nucleotide-binding oligomerisation domain (NOD) proteins, NOD1 and NOD2, two intracellular pattern-recognition molecules of the NLR (nucleotide-binding leucine-rich repeat receptor) family. The NOD1 and NOD2 proteins are intracellular receptors for bacterial peptidoglycans, and induce pro-inflammatory signalling and anti-microbial responses in immune cells [52]. Upon stimulation, displacement of HSPB8 from HRI to the NOD1 signalosome appears to trigger an HRI-dependent eIF2α-ATF4-ATF3 pathway, resulting in transcriptional up-regulation of HSPB8 to maintain cellular homeostasis [16]. In previously discussed work of Patel *et al.*, they also suggest that the presence of BAG3 would stimulate HRI degradation. Thus, the present study would suggest that HSPB8 down-regulates HRI, but this regulation is dependent on the BAG3/Hsp70 complex; thus the mechanism underlying this regulation remains unclear.

## Conclusions

In this review, we have tried to highlight the known regulators of HRI and how they might interact with HRI to mediate the ISR under a diverse range of stresses. What is apparent is that the lack of structural information for HRI, especially regarding how haem binding facilitates inhibition, makes subsequent insight into how other activators and inhibitors work difficult to discern. Given that inactive HRI almost certainly resides in the cytosol, bound to haem, understanding this baseline is conformation of HRI is critical, as it informs all subsequent regulatory events. Speculatively, it may be that there are at least two states/pools of HRI, which employ different mechanisms of regulation: a first (mitochondrial?) HRI pool where the SIFI complex is responsible for down-regulating HRI activated by DELE1^CTD^, and a second (cytoplasmic?) pool of HRI, bound to BAG3/Hsp70, which is down-regulated by HSPB8 binding. This model is almost certainly incomplete, as it does not account for haem-mediated regulation. Future research on how HRI can integrate these signals will be dependent on structural and cellular biochemical work working in tandem.

## Perspectives

- HRI is an important candidate for several pathologies, including cancer and neurodegeneration, and appears to play a significant role in managing proteostasis under several stresses.
- In the absence of a structure of HRI, several aspects of HRI regulation must be inferred from biochemistry and using structural prediction. While there appears to be robust evidence for haem-mediated inhibition of HRI, turnover of HRI by the SIFI complex, and activation by DELE1, even these regulatory mechanisms lack fundamental details.
- What is most required to accelerate our understanding of HRI are activated and inhibited (haem-bound) structures of the dimer. In lieu of this, a better understanding of HRI regulation by BAG3-Hsp70/HSPB, p58^IPK^, and DELE1^CTD^ could be achieved through *in vitro* biochemical assays with purified proteins produced from mammalian or eukaryotic expression systems rather than *E.coli*.

## Open Access

Open access for this article was enabled by the participation of the University of Dundee in an all-inclusive Read & Publish agreement with Portland Press and the Biochemical Society under an agreement with JISC.

## Abbreviations

HBD: haem-binding domain
HRI: haem-regulated inhibitor
ISR: integrated stress response
NOD: nucleotide-binding oligomerisation domain
SIFI: silencing factor of the integrated stress response
TPR: tetratricopeptide repeat
